# Dietary Microencapsulated Roselle (*Hibiscus sabdariffa* L.) Calyx Extract Enhances Growth, Pigmentation, and Physiological Responses in Fancy Carp (*Cyprinus carpio* L.)

**DOI:** 10.1155/anu/8985910

**Published:** 2026-06-29

**Authors:** Supalug Kattakdad, Nittaya Phungam, Suriya Udduang, Sophasith Thammaboud

**Affiliations:** ^1^ Department of Fisheries, Faculty of Agriculture and Technology, Rajamangala University of Technology Isan, Surin, Thailand, rmuti.ac.th; ^2^ Department of Agro-Industrial, Faculty of Agriculture and Technology, Rajamangala University of Technology Isan, Surin, Thailand, rmuti.ac.th; ^3^ Department of Livestock and Fisheries, Ministry of Agriculture and Environment, Vientiane, Lao PDR

**Keywords:** antioxidant, fancy carp, feed additive, functional feed, microencapsulation, ornamental fish, roselle

## Abstract

This study evaluated the optimal dietary inclusion level of microencapsulated roselle (*Hibiscus sabdariffa* L.) calyx extract (MRE) and its effects on growth, physiological responses, pigmentation, and tissue histology in fancy carp (*Cyprinus carpio* L.). Fish (27.09 ± 1.14 g) were randomly assigned to 500 L tanks (30 fish/tank) and fed diets containing 0% (MRE‐0), 1% (MRE‐1), 2% (MRE‐2), 3% (MRE‐3), and 4% (MRE‐4) MRE for 60 days, with three replicates per treatment. Fish fed MRE‐2, MRE‐3, and MRE‐4 showed significantly higher final weight (*p* = 0.001), weight gain (WG) (*p* = 0.003), specific growth rate (SGR) (*p* = 0.003), and average daily gain (ADG) (*p* = 0.003) than those fed MRE‐0 and MRE‐1, with MRE‐3 and MRE‐4 exhibiting the lowest feed conversion ratios (FCR) (*p* = 0.001). Polynomial regression showed a significant relationship between MRE level and SGR (*R* ^2^ = 0.9717), with the predicted optimum at 3.76%, which closely aligning with the observed results. No significant differences were found in survival rate (SR) (90%–100%), hepatosomatic and viscerosomatic indices. Hematological parameters showed significant increases in red blood cell (RBC) counts (*p* = 0.027), hemoglobin (*p* = 0.003), total protein (*p* = 0.033), and immunoglobulin levels (*p* = 0.001) in MRE‐3 and MRE‐4 groups. MRE supplementation significantly enhanced lysozyme (*p* = 0.008) and antioxidant enzyme activities (superoxide dismutase [SOD], *p* = 0.011; glutathione peroxidase [GPx], *p* = 0.014), with the highest values in MRE‐3 and MRE‐4. Redness (a^∗^) values significantly increased in MRE‐3 and MRE‐4‐fed fish, while lightness (L^∗^) and yellowness (b^∗^) remained unchanged. Carotenoid and anthocyanin levels in skin, fin, and muscle increased with higher MRE levels. Histologically, MRE‐3 improved intestinal villus height, whereas MRE‐4 induced more pronounced hepatic hyaline degeneration. In conclusion, dietary inclusion of 3% MRE was identified as the optimal level for fancy carp, improving growth, feed efficiency, immune and antioxidant responses, pigmentation, and intestinal development without adverse hepatic effects.

## 1. Introduction

Plant‐derived bioactive compounds have gained increasing attention in aquaculture as functional feed additives due to their potential to enhance growth, antioxidant status, immune responses, and disease resistance [1]. Polyphenols and other phytochemicals can modulate physiological and immune functions, thereby improving fish health and production performance [1]. Previous studies have demonstrated the beneficial effects of various medicinal plant‐derived additives on growth, immunity, antioxidant capacity, and stress resistance in several cultured fish species, highlighting their potential as sustainable alternatives to synthetic feed additives in aquaculture [2–7]. Among these medicinal plants, roselle (*Hibiscus sabdariffa* L.) has emerged as a promising functional ingredient owing to its rich phytochemical profile and diverse health‐promoting properties. Various parts of the plant contain different bioactive compounds, making it useful in traditional medicine, pharmaceuticals, functional foods, and the nutraceutical industry [8–10]. The fruits are rich in vitamin C, fatty acids, and essential minerals, while the seeds are a significant source of vitamin E, fatty acids, and minerals [11, 12]. The leaves are characterized by alcohol derivatives, vitamin E derivatives, and dietary fiber, and the roots are noted for their high concentrations of saponins and other bioactive compounds [12]. The calyx is rich in organic acids, phenolic compounds, flavonoids, and anthocyanins, which contribute to its strong antioxidant, antimicrobial, and pigmentation‐enhancing activities [10, 12]. These compounds exhibit antioxidant properties by neutralizing reactive oxygen species, chelating pro‐oxidant metals, and inhibiting radical‐generating enzymes [11, 13, 14]. In living organisms, oxidative stress is counteracted by complex antioxidant defense systems, which include both enzymatic components (e.g., superoxide dismutase [SOD], catalase, and peroxidases) and nonenzymatic factors that work together to maintain cellular homeostasis [14, 15]. Additionally, roselle is a rich source of pigments such as anthocyanins and carotenoids, making it a suitable natural additive for improving coloration in ornamental fish [12, 16]. Its functional properties support its use as a natural preservative and health enhancer in both food and aquafeed formulations [9]. In aquaculture, roselle has shown promising effects in several species. Fermented roselle seed meal has been tested as a plant protein alternative in *Clarias gariepinus* diets [17]. In Nile tilapia, supplementation with roselle calyx improved growth, feed efficiency, and resistance to *Aeromonas hydrophila* through enhanced lysozyme activity and bactericidal activities [18]. In goldfish (*Carassius auratus*), roselle‐derived anthocyanins promoted growth and skin pigmentation [19, 20]. Similarly, in rainbow trout (*Oncorhynchus mykiss*), roselle extract improved immune responses, alleviated stress, and modulated cytokine expression [21]. These findings suggest that roselle calyx is a promising natural feed additive for improving fish health, physiological performance, and product quality in aquaculture.

Fancy carp (*C. carpio* L.), a member of the Cyprinidae family and commonly referred to as ornamental koi, is highly valued for its vibrant coloration, making it a popular species in ornamental aquaculture [22]. High‐quality fancy carp, characterized by distinct breed‐specific traits and vivid coloration, can be worth several 1000 US dollars per individual. Due to their significant market demand and economic importance, selective breeding programs have been established to produce strains with stable genetic diversity and consistent inheritance of body color traits [23]. However, a common challenge in fancy carp culture is the progressive fading of coloration over time, which diminishes their market value [24]. In addition to genetic improvements, enhancing growth, immune function, and coloration through natural feed additives has gained considerable attention in ornamental aquaculture [25–29]. Despite the reported benefits of roselle‐derived additives, their practical application is limited by thermal degradation during feed processing, particularly during the production of floating pelleted feeds where temperatures can reach up to 140°C [30], leading to a continuous decline in key bioactive compounds such as anthocyanins at temperatures up to 110°C [31]. This limitation reduces the effectiveness of additives in improving fish coloration and health. To address this challenge, microencapsulation has been employed as a protective strategy to maintain the stability and bioavailability of roselle‐derived bioactive compounds during feed processing, providing a novel approach to enhance the functional efficacy of ornamental fish diets.

Microencapsulation involves enclosing solid, liquid, or gaseous substances within microcapsules, enabling controlled, sustained release over time [32]. This technique enhances the stability and bioavailability of active compounds by embedding them in a protective matrix [33], thereby extending their shelf life and functionality in various applications, including aquaculture [34–38]. Methods such as emulsification‐crosslinking, spray drying, and coacervation have proven effective in preserving the functional integrity of encapsulated substances [39]. Given the promising potential of roselle in aquaculture, particularly for ornamental fish production, this study aimed to determine the optimal dietary level of microencapsulated roselle calyx extract for fancy carp (*C. carpio* L.). The research focused on evaluating its effects on growth performance, hematological parameters, color expression, pigment accumulation, and tissue histology, with the goal of providing insights into the application of microencapsulated plant bioactives for enhancing nutrient utilization and supporting sustainable feed strategies in ornamental aquaculture.

## 2. Materials and Methods

### 2.1. Experimental Plan

A completely randomized design was employed to evaluate the effects of dietary supplementation with microencapsulated roselle extract (MRE) at five concentrations: 0% (MRE‐0), 1% (MRE‐1), 2% (MRE‐2), 3% (MRE‐3), and 4% (MRE‐4). Each treatment included three independent replicates, with 30 fish per replicate in separate tanks. These supplementation levels were informed by the study of Jomeh et al. [21], which investigated the application of anthocyanin extract from roselle calyx in aquafeeds.

### 2.2. Experimental Diets Preparation

**Figure 1:**
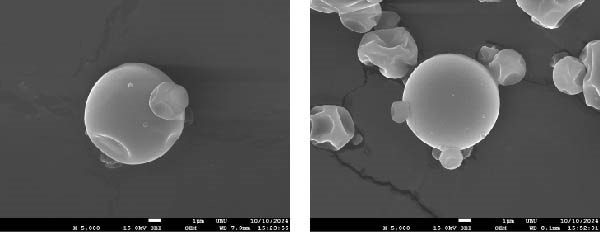
Scanning electron micrograph of MRE prepared by spray‐drying, observed at 5000× magnification and 15.0 kV.

The calyx of roselle was extracted using water at a 1:2 (w/v) ratio of dried calyx to water, followed by homogenization until a fine consistency was achieved. The mixture was then covered and left to stand at 25°C for 24 h. After the extraction period, the sample was filtered using number 1 filter paper to separate the residual solids from the roselle extract. The crude extract was mixed with 5% (w/v) maltodextrin as the encapsulating agent. The mixture was stirred thoroughly to ensure homogeneity and complete dissolution in distilled water. The encapsulation was performed using a laboratory‐scale spray dryer (Model SDE‐10, Eyela, Japan). The solution was fed into the spray dryer at a constant flow rate of 5 mL min^−1^. The drying process was conducted at an inlet air temperature of 130°C, and the outlet temperature was maintained between 80°C and 90°C. These parameters were optimized to ensure efficient drying without compromising the bioactive components of the extract. The resulting encapsulated powder was collected from the cyclone separator and stored in airtight containers at 4°C with low humidity (<10%) for up to 1 week prior to feed incorporation to maintain the stability of the bioactive compounds. The characteristics of the microencapsulated crude extract from roselle are illustrated in Figure 1. The bioactive compounds of MRE were assessed as follows: total carotenoids were determined using the method of Gogoi et al. [40], total anthocyanins were analyzed following the method of Fuleki and Francis [41], total flavonoid content was determined using the aluminum chloride colorimetric method [42], and total phenolic compounds were measured using the Folin‐Ciocalteu method [43]. The results of the bioactive compounds of MRE are presented in Table 1.

**Table 1:** Bioactive compounds of microencapsulated roselle extract.

Bioactive compounds	Amounts
Total carotenoid (mg 100 g^−1^)	0.52 ± 0.07
Total anthocyanins content (mg CGE 100 g^−1^)	48.65 ± 0.11
Total flavonoids content (mg CTE 100 g^−1^)	583.79 ± 5.52
Total phenolic content (mg GAE 100 g^−1^)	76.83 ± 0.16

*Note:* Values are presented as mean ± standard deviation (*n* = 3).

Abbreviations: CGE, cyanidin‐3‐glucoside equivalents; CTE, catechin equivalents; GAE, gallic acid equivalents.

The raw feed ingredients were ground, sieved through a 320‐µm mesh, and blended according to the experimental diet formulations (Table 2). The experimental diets were supplemented with MRE at five inclusion levels (0%, 1%, 2%, 3%, and 4%) according to the experimental design. The mixtures were then processed into pellets using a single‐screw extruder machine operated at a barrel temperature of 120°C and a pressure of ~4 bar to ensure proper expansion and pellet texture while minimizing nutrient loss. Following pellet formation, the pellets were dried in a hot air oven at 65°C for 12 h. After drying, the diets were stored in airtight containers at 4°C until use. The nutritional composition of the experimental diets was analyzed using the methods of AOAC [44], with the results presented in Table 2.

**Table 2:** Formulations and chemical composition of experimental diets with graded levels of MRE (% dry weight).

Ingredients	Experimental diets				
	MRE‐0	MRE‐1	MRE‐2	MRE‐3	MRE‐4
Fish meal (P60)	23	23	23	23	23
Soybean meal	31	31	31	31	31
Defatted rice bran	15	15	15	15	15
Corn meal	15	15	15	15	15
Cassava starch	10	9	8	7	6
MRE^a^	0	1	2	3	4
Soybean oil	1	1	1	1	1
Fish oil	1	1	1	1	1
Di‐calcium phosphate	1	1	1	1	1
Vitamin and mineral premix	1	1	1	1	1
Vitamin C	1	1	1	1	1
Binder	1	1	1	1	1

Chemical composition by proximate analysis^b^ (% dry weight basis)					
Crude protein	32.31 ± 0.31	32.29 ± 0.64	32.28 ± 0.77	32.25 ± 0.52	32.24 ± 0.46
Crude fat	5.72 ± 0.03	5.77 ± 0.09	5.63 ± 0.08	5.68 ± 0.06	5.69 ± 0.05
Crude fiber	5.14 ± 0.09	5.05 ± 0.06	5.04 ± 0.06	5.04 ± 0.07	5.03 ± 0.09
Crude ash	9.23 ± 0.18	9.21 ± 0.12	9.22 ± 0.05	9.23 ± 0.05	9.25 ± 0.03
Moisture content	8.67 ± 0.06	9.00 ± 0.13	8.96 ± 0.11	8.82 ± 0.08	8.74 ± 0.09
Values by calculation					
Nitrogen‐free extracts^c^ (%)	38.93 ± 0.40	38.68 ± 0.31	38.87 ± 1.01	38.98 ± 0.69	39.05 ± 0.46

^a^Microencapsulated roselle extract.

^b^Values are presented as mean ± SD (*n* = 3).

^c^Nitrogen‐free extract = 100 − (moisture + crude protein + crude lipid + crude fiber + crude ash).

### 2.3. Fish Feeding and Management

Fancy carp (*C. carpio* L.) were sourced from the Surin Inland Fisheries Research and Development Center, Surin Province, Thailand. Prior to the experiment, fish were acclimated to the culture conditions for 1 week. The study was conducted in 500‐liter plastic tanks, each containing 300 liters of water. Healthy fish with species‐appropriate characteristics (initial mean body weight of 27.09 ± 1.14 g and initial mean total length of 7.78 ± 0.44 cm) were randomly distributed into 15 tanks at a stocking density of 30 fish per tank (2.71 g fish L^−1^). The tanks were then randomly allocated to five dietary treatments (three tanks per treatment). Fish were fed three times daily (8:00 a.m., 12:00 p.m., and 4:00 p.m.) to satiation. The feeding trial lasted for 60 days, during which fish behavior, feeding responses, and clinical signs were monitored to prevent diseases. Sediment was removed, and ~20% of the water volume in each tank was replaced weekly to maintain water quality. Water temperature and dissolved oxygen (DO) were measured twice daily (8:00 a.m. and 15:00 p.m.) using a handheld multiparameter meter (Hanna Instruments, Model HI 98193; Rhode Island, USA). The pH was monitored twice daily (8:00 a.m. and 15:00 p.m.) using a pH meter (Hanna Instruments, Model HI 98191; Rhode Island, USA). Total ammonia–nitrogen (TAN) was determined twice weekly using the phenate spectrophotometric method following APHA [45]. Throughout the experiment, water temperature was maintained at 28–29°C, pH remained within 7.8–8.5, DO levels were consistently above 5 mg L^−1^, and TAN concentrations were kept below 0.02 mg L^−1^ to ensure optimal water quality for ornamental carp.

Throughout the study, all fish and experimental procedures were conducted in strict accordance with the guidelines for the Care and Use of Animals for Scientific Purposes as outlined by the National Research Council of Thailand (NRCT), Thailand (Number U1‐05537−2559).The experimental protocol was approved by the Institutional Animal Care and Use Committee, ensuring compliance with the ethical standards set by the NRCT for the use of animals in scientific research (Certificate of Approval ID: 03–66−002). All procedures were conducted in accordance with the 3Rs principles to limit animal use and uphold high standards of animal welfare.

### 2.4. Growth, Feed Utilization, and Somatic Indices

Fish were starved for 24 h prior to sampling, and total feed intake was recorded. At the conclusion of the experiment, the weight of all fish was measured using an electronic balance with a precision of 0.1 g. Growth performance, feed utilization, and somatic indices were evaluated based on the following parameters: weight gain (WG), average daily gain (ADG), specific growth rate (SGR), feed conversion ratio (FCR), survival rate (SR), hepatosomatic index (HSI), and viscerosomatic index (VSI). The growth performance parameters were calculated according to Saripan et al. [46] using the following equations:

WG % Fish−1=Final weight −Initial weight/Initial weight × 100,

ADG g day−1 =Final weight −Initial weight/60 days,

SGR % day−1 =Ln final weight−Ln Initial weight × 100/60 days,

FCR =Amount of feed given g/Weight gain g,

SR %=No. of fish harvested/No. of fish stocked × 100,

HSI %=Liver weight g/Body weight g × 100,

VSI %=Viscera weight g/Body weight g × 100.

### 2.5. Hematological Parameters

Fish were fasted for 24 h, and blood sampling was performed between 8:00 and 10:00 a.m. to control postprandial and circadian variations in hematological parameters. All blood samples were collected from the caudal vein of five fish per tank using a syringe equipped with a number 24 hypodermic needle, following anesthesia with clove oil at a concentration of 30 ppm. Samples were mixed with an anticoagulant (EDTA, 1.5 mg mL^−1^ blood) to determine total red blood cell (RBC) and white blood cell (WBC) counts using Dacie’s fluid reagent as described by Blaxhall and Daisley [47]. Hemoglobin (Hb) levels were measured using the cyanmethemoglobin method. Additional blood samples were collected without an anticoagulant and allowed to coagulate at 4°C for 15 min. The samples were then centrifuged at 9000 × *g* for 10 min at 4°C to separate the serum, which was then used for the analysis of total protein content following the method of Lowry et al. [48] and total immunoglobulin levels according to Siwicki et al. [49]. Lysozyme activity was assessed using ELISA assay kits (Product Number LY0100–1 KT, Sigma–Aldrich, USA) at 450 nm with a detection limit of 0.01 U mL^−1^. SOD activity (Product Number 19160, Sigma–Aldrich, USA) was measured at 450 nm with a detection limit of 0.01 U mL^−1^, and glutathione peroxidase (GPx) activity (Product Number MAK437, Sigma–Aldrich, USA) was determined at 412 nm with a detection limit of 0.03 U mL^−1^. Enzymatic assays were performed in triplicate and normalized to milligrams of protein from the same homogenate.

### 2.6. Color Expression

Five fish from each tank were selected to evaluate body color expression using a color space Common Intermediate Format (CIF) system with a Color Reader Model, CR10 (Konica Minolta Sensing, INC., Japan). Fish were anesthetized with clove oil (30 ppm) and gently blotted to remove excess water. The instrument was calibrated before each session using standard white and black reference points to measure lightness (L^∗^), redness (a^∗^), and yellowness (b^∗^). Color measurements were recorded on the fish’s body, specifically below the dorsal fin and above the lateral line.

### 2.7. Pigment Accumulation

Samples of viscera, fillet, fin, skin, and scales were collected from five fish per tank following anesthesia with clove oil (30 ppm) for pigment accumulation analysis. Tissue samples were homogenized using a hand‐held homogenizer (MT‐30 K, Hangzhou Miu Instruments Co., Ltd., China) at 22,000 rpm for 2 min under cold conditions to prevent the degradation of bioactive compounds. Carotenoids were extracted using chloroform as the extraction solvent, following the method of Gogoi et al. [40]. Total anthocyanin content was determined according to the method of Fuleki and Francis [41], using ethanol–1.5 N hydrochloric acid (85:15, v/v) as the extraction solvent.

### 2.8. Tissue Histology

The middle intestine and liver of five fish from each tank were collected for histological analysis, following standard procedures as described by Peng et al. [50]. Tissue samples were fixed in 10% neutral buffered formalin for 24 h, embedded in paraffin, sectioned at 5 µm thickness, and stained with hematoxylin and eosin (H&E) for general histological evaluation. Digital images of tissue sections were acquired using an Olympus CX33 light microscope equipped with a Canon EOS750D camera and operated via EOS utility software. For intestinal morphology, 10 well‐oriented villi per section were randomly selected for measurement of villus height and width as well as goblet cell count, and the mean values were used for statistical analysis. Histological observations were primarily qualitative, providing a descriptive evaluation of tissue structure and cellular integrity.

### 2.9. Data Analysis

Mean values and standard deviations (S.D.) were calculated from the raw data, with each tank considered an independent experimental unit. Data analyses were conducted using tank means, and homogeneity of variances was confirmed by Levene’s test prior to analysis. Differences among treatment groups were assessed using one‐way ANOVA, followed by Duncan’s multiple range test (DMRT) for post‐hoc comparisons when significant differences were observed (*p* ≤ 0.05). Polynomial regression analysis was performed to describe the relationship between dietary MRE levels and growth performance parameters (SGR). Model adequacy was verified by examining the residual plots for normality and homoscedasticity. Fitted regression curves are presented with 95% confidence intervals to illustrate the precision of the model.

## 3. Results

### 3.1. Growth, Feed Utilization, and Somatic Indices

Growth performance, feed utilization, SR, and somatic indices of fancy carp (*C. carpio* L.) after 60 days of feeding with experimental diets are shown in Table 3. At the end of the experiment, fish fed diets supplemented with MRE‐2, MRE‐3, and MRE‐4 exhibited significantly higher final body weights compared to those fed MRE‐1 and the control diet (MRE‐0) (*p* = 0.001). Notably, the MRE‐2, MRE‐3, and MRE‐4 groups exhibited statistically similar values for mean body weight, WG, ADG, and SGR. However, these values were significantly higher than those observed in the MRE‐1 and MRE‐0 groups. The results of feed utilization, in terms of FCR, indicated that fish fed MRE‐3 and MRE‐4 had significantly lower FCR values (*p* = 0.001) compared to those fed MRE‐2, MRE‐1, and MRE‐0, while feed intake did not differ significantly among treatments. Meanwhile, the SR across all experimental groups did not differ significantly (*p* = 0.398), with values ranging from 90% to 100%. Similarly, no significant differences were observed in the somatic indices, including the HSI (*p* = 0.237) and VSI (*p* = 0.314).

**Table 3:** Growth performance, feed utilization, SR, and somatic indices of fancy carp (*C. carpio* L.) after 60 days of feeding with experimental diets.

Parameters	Experimental diets					*p*‐Value
	MRE‐0	MRE‐1	MRE‐2	MRE‐3	MRE‐4	
Initial body weight (g)	27.35 ± 0.06	27.12 ± 1.68	26.82 ± 1.20	27.03 ± 1.55	27.11 ± 1.23	0.990
Final body weight (g)	48.45 ± 1.01^b^	50.31 ± 1.86^b^	53.64 ± 1.45^a^	55.57 ± 1.91^a^	55.41 ± 1.67^a^	0.001
WG (%)	77.12 ± 3.35^b^	85.95 ± 12.55^b^	100.18 ± 6.20^a^	105.87 ± 8.43^a^	104.48 ± 3.40^a^	0.003
ADG (g day^−1^)	1.28 ± 0.06^b^	1.43 ± 0.21^b^	1.67 ± 0.11^a^	1.76 ± 0.14^a^	1.74 ± 0.06^a^	0.003
SGR (% day^−1^)	0.95 ± 0.03^b^	1.03 ± 0.12^b^	1.16 ± 0.05^a^	1.20 ± 0.07^a^	1.19 ± 0.03^a^	0.003
FCR	2.73 ± 0.06^a^	2.61 ± 0.17^ab^	2.47 ± 0.03^b^	2.28 ± 0.08^c^	2.29 ± 0.08^c^	0.001
SR (%)	96.67 ± 5.77	96.67 ± 5.77	90.00 ± 10.00	100 ± 0.00	90.00 ± 10.00	0.398
Somatic indices						
HSI (%)	3.92 ± 0.39	2.94 ± 0.39	3.45 ± 0.23	3.65 ± 0.30	3.54 ± 0.86	0.237
VSI (%)	6.48 ± 1.45	8.07 ± 0.83	7.77 ± 0.69	7.38 ± 2.07	6.08 ± 0.58	0.314

*Note:* Values are expressed as mean ± SD. Different superscripts within the same row denote significant differences at *p* ≤ 0.05.

Abbreviations: ADG, average daily gain; FCR, feed conversion ratio; HSI, hepatosomatic index; SGR, specific growth rate; SR, survival rate; VSI, viscerosomatic index; WG, weight gain.

**Figure 2:**
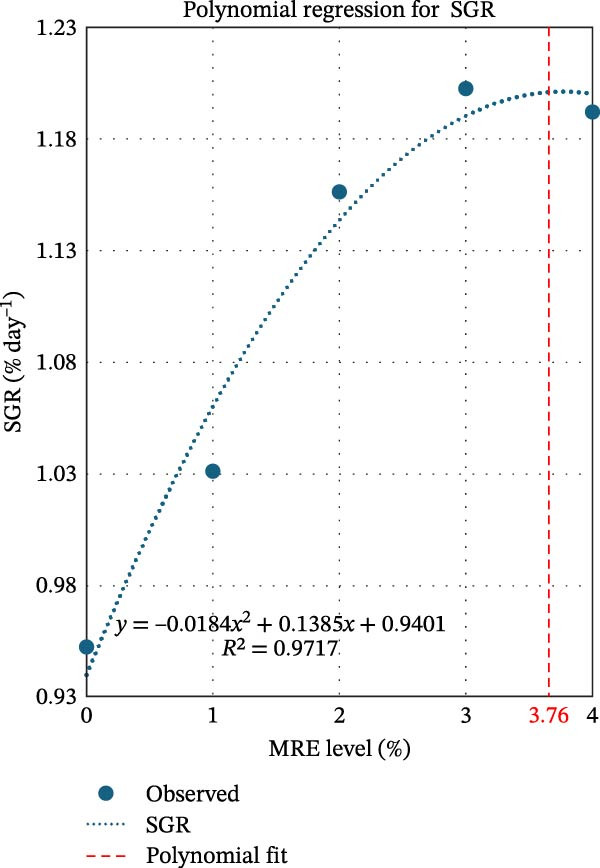
Polynomial regression analysis between dietary MRE supplementation levels and SGR of fancy carp (*C. carpio* L.) with 95% confidence intervals.

Polynomial regression analysis indicated a significant relationship between dietary MRE supplementation and SGR of fancy carp, as described by the equation: SGR = −0.0184x^2^ + 0.1385x + 0.9401 (*R* ^2^ = 0.9717). The high coefficient of determination (*R* ^2^ = 0.9717) indicates that 97.17% of the variation in SGR was explained by the MRE supplementation level. Based on the regression model, the predicted optimal inclusion level of MRE for maximizing SGR was 3.76%, at which point the highest SGR was observed, as shown in Figure 2.

### 3.2. Hematological Parameters

As shown in Table 4, the hematological parameters revealed that fish fed diets supplemented with MRE‐3, MRE‐4, and MRE‐2 exhibited significantly higher RBC counts compared to those fed MRE‐0 diet (*p* = 0.027). Conversely, WBC counts did not differ significantly among the experimental groups (*p* = 0.633). Fish fed MRE‐3 and MRE‐4 diets demonstrated significantly higher Hb levels compared to those in the MRE‐2, MRE‐1, and MRE‐0 groups (*p* = 0.003). Additionally, total protein levels were significantly elevated in fish fed MRE‐3, MRE‐4, and MRE‐2 diets relative to the MRE‐0 group (*p* = 0.033). These findings were consistent with the total immunoglobulin levels, which were significantly higher in fish fed MRE‐3 and MRE‐4 diets compared to other experimental groups (*p* = 0.001). The analysis of lysozyme activity revealed a positive correlation with the increased supplementation of MRE. Fish fed the MRE‐4 diet exhibited the highest lysozyme activity among all experimental groups (*p* = 0.008). The analysis of SOD activity revealed that fish fed MRE‐3 and MRE‐4 diets exhibited significantly higher SOD levels compared to those in the MRE‐1 and MRE‐0 groups (*p* = 0.011). Similarly, the evaluation of GPx activity indicated that fish fed the MRE‐3 diet achieved the highest GPx levels among all treatments (*p* = 0.014).

**Table 4:** Hematological parameters of fancy carp (*C. carpio* L.) after 60 days of feeding with experimental diets.

Parameters	Experimental diets					*p*‐Value
	MRE‐0	MRE‐1	MRE‐2	MRE‐3	MRE‐4	
RBC (10^6^ cell µL^−1^)	1.82 ± 0.05^b^	1.97 ± 0.18^ab^	2.07 ± 0.05^a^	2.13 ± 0.09^a^	2.08 ± 0.09^a^	0.027
WBC (10^3^ cell µL^−1^)	1.29 ± 0.51	1.54 ± 0.30	1.53 ± 0.50	1.81 ± 0.25	1.62 ± 0.33	0.633
Hb (g dL^−1^)	10.22 ± 0.50^c^	10.80 ± 0.81^bc^	11.70 ± 0.31^ab^	12.25 ± 0.46^a^	12.02 ± 0.34^a^	0.003
Total protein (g dL^−1^)	9.42 ± 0.41^b^	10.94 ± 1.32^ab^	11.74 ± 1.25^a^	12.30 ± 0.84^a^	12.10 ± 0.95^a^	0.033
Total immunoglobulin (g dL^−1^)	5.12 ± 0.85^c^	5.24 ± 0.37^c^	6.30 ± 0.62^b^	7.57 ± 0.31^a^	7.42 ± 0.51^a^	0.001
Lysozyme activity (µg mL^−1^)	11.71 ± 0.60^c^	12.47 ± 0.54^bc^	12.81 ± 0.25^ab^	13.04 ± 0.27^ab^	13.33 ± 0.34^a^	0.008
SOD (U mg^−1^ protein)	4.37 ± 0.45^b^	4.86 ± 0.74^b^	5.35 ± 0.38^ab^	6.14 ± 0.66^a^	6.06 ± 0.39^a^	0.011
GPx (U mg^−1^ protein)	5.09 ± 0.15^c^	6.00 ± 1.12^bc^	5.90 ± 0.29^bc^	7.85 ± 0.90^a^	7.05 ± 1.02^ab^	0.014

*Note:* Values are expressed as mean ± SD. Different superscripts within the same row denote significant differences at *p* ≤ 0.05.

Abbreviations: GPx, glutathione peroxidase; Hb, hemoglobin; RBC, red blood cells; SOD, superoxide dismutase; WBC, white blood cells.

### 3.3. Color Expression

**Figure 3:**
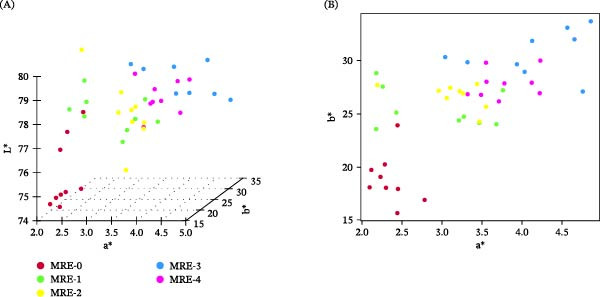
Color expression of fancy carp (*C. carpio* L.) after 60 days of feeding with the experimental diets. (A) Color distribution of fancy carp based on L^∗^, a^∗^, and b^∗^ values. (B) Color distribution of fancy carp based on bivariate 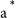 and 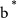 values.

The color expression of fancy carp is shown in Table 5 and Figure 3. Lightness (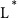) values on the body of fish across all experimental groups did not differ significantly (*p* = 0.268), with values ranging from 75.77 to 78.65. However, the redness (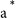) values of fish fed the MRE‐3 and MRE‐4 diets were significantly higher compared to other experimental groups (*p* = 0.035). Conversely, the yellowness (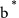) values of fish fed the control diet (MRE‐0) were the lowest (*p* = 0.010), while no significant differences were observed among the MRE‐supplemented groups.

**Table 5:** Color expression of fancy carp (*C. carpio* L.) after 60 days of feeding with the experimental diets.

Parameters	Experimental diets					*p*‐Value
	MRE‐0	MRE‐1	MRE‐2	MRE‐3	MRE‐4	
Lightness (L^∗^)	75.77 ± 1.74	78.23 ± 0.36	77.63 ± 2.50	78.65 ± 1.15	77.78 ± 1.15	0.268
Redness (a^∗^)	2.44 ± 0.34^b^	2.68 ± 087^b^	2.92 ± 0.66^b^	4.31 ± 0.82^a^	3.68 ± 0.48^ab^	0.035
Yellowness (b^∗^)	19.63 ± 3.76^b^	25.48 ± 2.93^a^	26.48 ± 1.93^a^	30.24 ± 3.30^a^	26.85 ± 0.10^a^	0.010

*Note:* Values are expressed as mean ± SD. Different superscripts within the same row denote significant differences at *p* ≤ 0.05.

### 3.4. Pigment Accumulation

**Figure 4:**
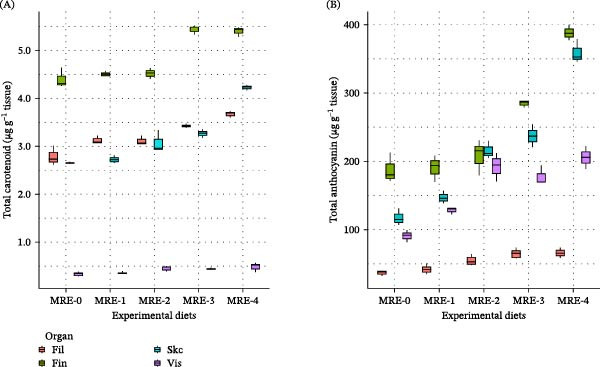
Total carotenoid (A) and anthocyanin (B) accumulation in various organs of fancy carp (*C. carpio* L.) after 60 days of feeding with the experimental diets. Abbreviations: fil, fillet; fin, fin; skc, skin and scale; vis, viscera.

The pigment accumulation in fancy carp after 60 days of feeding with the experimental diets is shown in Figure 4. The results indicated that the highest accumulation of carotenoids occurred in the fins, followed by similar levels in the fillet, skin, and scales. The lowest carotenoid accumulation was observed in the viscera. When the accumulation of carotenoids in fish fed diets supplemented with varying concentrations of MRE was compared, it was found that the amount of supplementation was positively correlated with the carotenoid accumulation. Similarly, the anthocyanin accumulation in fancy carp was found to vary according to the amount of MRE supplemented in the diet. However, it was observed that anthocyanin accumulation in the fins, skin, and scales was similar across all experimental groups, while accumulation in the viscera was higher than that in the fillet tissue.

### 3.5. Tissue Histology

**Figure 5:**
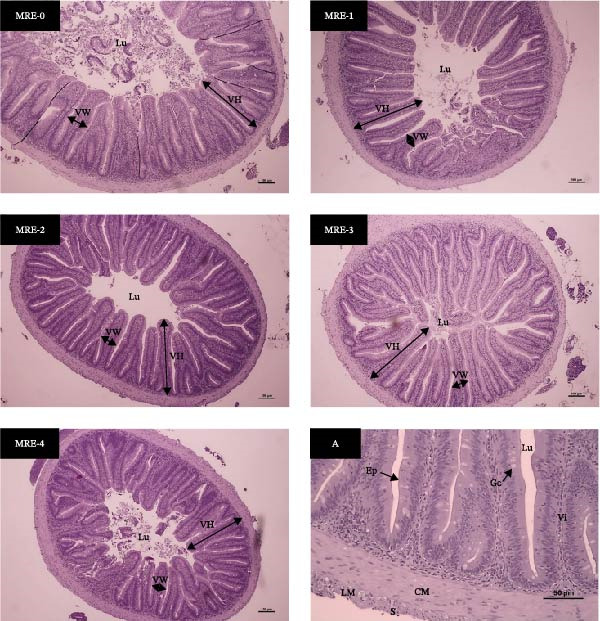
Microscopic examination of intestinal histology in fancy carp (*C. carpio* L.) after 60 days of feeding with experimental diets. Images were captured at 10× magnification. The inset (Image A), observed at 40× magnification, shows the detailed intestinal structures, including the serosa (S), longitudinal muscle (LM), circular muscle (CM), goblet cells (GC), villi (Vi), villus height (VH), villus width (VW), and epithelial cells (Ep).

Figure 5 presents representative histological cross‐sections of intestinal tissue in fancy carp after 60 days of feeding with the experimental diets. Among all treatment groups, fish receiving the MRE‐3 diet exhibited a significantly greater villus height (*p* = 0.002), indicating improved intestinal development. Conversely, no significant differences in villus height were observed among the remaining groups. Interestingly, the MRE‐3 group showed the lowest villus width (*p* = 0.023), while no significant differences were observed in goblet cell counts among dietary treatments (*p* = 0.174), as shown in Table 6.

**Table 6:** Villus height, villus width, and goblet cell count in the intestine of fancy carp (*C. carpio* L.) after 60 days of feeding with experimental diets.

Parameters	Experimental diets					*p*‐Value
	MRE‐0	MRE‐1	MRE‐2	MRE‐3	MRE‐4	
Villus height (µm)	1290.42 ± 100.53^b^	1290.69 ± 113.88^b^	1329.99 ± 69.23^b^	1517.74 ± 65.11^a^	1291.90 ± 77.87^b^	0.002
Villus width (µm)	194.51 ± 12.07^ab^	188.73 ± 19.05^ab^	209.76 ± 15.37^a^	173.97 ± 16.27^b^	208.17 ± 22.48^a^	0.023
Goblet cell count	19.40 ± 3.21	23.40 ± 2.97	21.60 ± 3.43	25.40 ± 5.77	22.20 ± 2.28	0.174

*Note:* Values are expressed as mean ± SD. Different superscripts within the same row denote significant differences at *p* ≤ 0.05.

**Figure 6:**
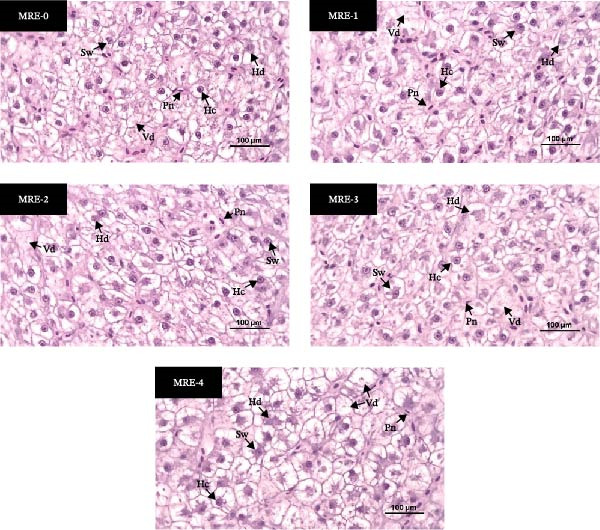
Histopathological changes in the liver of fancy carp (*C. carpio* L.) after 60 days of feeding with experimental diets. Micrographs were taken at 100× magnification, showing hepatocytes (Hc), pyknotic nuclei (Pn), vacuolar degeneration (Vd), hydropic swelling (Sw), and hyaline degeneration (Hd).

Histological analysis of the liver (Figure 6) revealed varying degrees of tissue alterations among the experimental groups. The hepatic parenchyma primarily consisted of hepatocytes with centrally located round nuclei and granular cytoplasm arranged in an ill‐defined architectural pattern. Histopathological changes observed across all treatment groups included pyknotic nuclei, vacuolar degeneration, hydropic swelling, and hyaline degeneration. Notably, fish in the MRE‐4 group exhibited more pronounced and widespread hyaline degeneration compared to other groups.

## 4. Discussion

This study demonstrated that dietary supplementation with MRE, rich in bioactive compounds such as phenolics, flavonoids, and anthocyanins, effectively enhanced growth performance, immunity, and physiological responses in fancy carp (*C. carpio* L.). A supplementation level of 3% (MRE‐3) was identified as optimal, producing superior growth performance and feed utilization efficiency without negatively affecting SR. Feed intake was consistent across treatments, indicating that MRE did not compromise palatability. The observed optimal growth performance closely aligned with the polynomial regression‐predicted optimum of 3.76% (*R* ^2^ = 0.9717), supporting 3% MRE as a practical and effective inclusion level that integrates both statistical and biological considerations. These findings align with previously published studies, for example, El Mesallamy et al. [51] demonstrated that supplementation with phenolic extracts derived from licorice roots significantly enhanced the growth performance of Nile tilapia (*Oreochromis niloticus*). Similarly, Linh et al. [52] reported that anthocyanin extracts from black rice bran significantly improved growth performance and immune responses in Nile tilapia cultivated in a biofloc system. Vanegas‐Espinoza et al. [20] also observed positive effects on growth and pigmentation in fantail goldfish (*Carassius auratus*) fed diets supplemented with microencapsulated anthocyanins derived from roselle. Additionally, Pérez‐Escalante et al. [19] found that supplementing goldfish (*C. auratus*) diets with anthocyanin extracts from roselle calyx flour improved SR and growth performance. However, contrasting results have been observed in some studies. Yilmaz [53] found no significant improvement in the growth performance of Nile tilapia fed diets supplemented with anthocyanin. Likewise, Jomeh et al. [21] reported no significant growth benefits in rainbow trout (*Oncorhynchus mykiss*) when fed diets containing anthocyanin extract from roselle. Moreover, excessive supplementation of anthocyanins can have negative effects on growth. Vanegas‐Espinoza et al. [20] reported that the inclusion of microencapsulated anthocyanins derived from roselle in fantail goldfish diets at high levels (450 mg kg^−1^) resulted in reduced growth performance. These discrepancies highlight the variability in responses to dietary bioactive compounds such as phenolics, anthocyanins, and flavonoids, which may be influenced by species‐specific physiology, culture conditions, and the bioavailability of the supplemented compounds.

Phenolic compounds, including benzoic acid, hydroxycinnamic acids, coumarins, flavonoids, anthraquinones, and anthocyanin, are diverse plant‐derived metabolites with significant antioxidant properties [54]. Flavonoids, comprising nearly two‐thirds of all phenolics, include anthocyanins as a key subgroup [55]. These compounds scavenge free radicals, prevent oxidative damage to cellular components, and chelate transition metals to limit free radical formation [56, 57]. They also enhance endogenous antioxidant enzymes (SOD, CAT, and GPx), stabilize lipid radicals, and protect cell membranes [58]. In MRE‐supplemented diets, phenolics improve fish health by enhancing hematological and immunological parameters. Increased RBC counts indicate better oxygen transport, while higher WBC levels in MRE‐3 suggest improved immune readiness. Elevated Hb, total protein, immunoglobulin, and enhanced lysozyme, SOD, and GPx activities in the MRE‐3 and MRE‐4 groups highlight MRE’s antioxidant and immunostimulatory effects, mitigating oxidative stress and enhancing resilience.

These findings align with studies on European sea bass (*Dicentrarchus labrax*), where phytophenol‐enriched diets from red grape extract improved immune responses in intestinal and splenic tissues [59], and on Nile tilapia, where black rice anthocyanin extract (4–8 g kg^−1^) stimulated growth and increased immune‐ and antioxidant‐related mRNA expression [44]. Roselle anthocyanin extract also increased RBC, Hb, hematocrit levels (Hct), and lysozyme activity while reducing cortisol in rainbow trout without affecting total protein or WBC [21]. Anthocyanins’ antioxidant properties protect RBCs from oxidative damage and hemolysis [60], and similarly, dietary supplementation with blackberry or roselle anthocyanin extracts enhanced Hct and antioxidant parameters in Nile tilapia [18, 53]. Furthermore, flavonoids share structural similarity with steroid hormones like estradiol, allowing binding to cellular receptors and activation of EGF/EGFR pathways, promoting cell division, tissue regeneration, and inhibiting apoptosis [61]. Specific flavones also support liver function by reducing plasma alanine aminotransferase (ALT), aspartate aminotransferase (AST), and lactate dehydrogenase (LD) levels while exhibiting antifungal, antiviral, and antibacterial properties [62, 63]. These observations are further supported by dietary phytogenic studies; Mohammady et al. [64] showed that lemon, onion, and garlic (LOG) at 20 mL kg^−1^ improved growth, feed efficiency, survival, enzyme activities, hematology, and key gene expression in Nile tilapia, and Gruber et al. [65] reported that matrix‐encapsulated phytogenics (Digestarom P.E.P. MGE) enhanced growth, immunity, and survival in fish challenged with *Streptococcus agalactiae*, highlighting the benefits of microencapsulated phytogenic compounds.

In this study, the MRE utilized contained anthocyanins and carotenoids, both of which are potent antioxidants crucial for pigmentation in aquafeeds. Higher supplementation levels of MRE were shown to enhance color expression (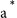 and 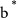 values) in fancy carp. This improvement suggests that the bioactive compounds, including anthocyanins and carotenoids, are effectively protected by microencapsulation during feed processing, enhancing their stability, gastrointestinal absorption, and tissue deposition, thereby promoting optimal pigmentation and nutrient utilization. Similar color enhancement has been observed with natural pigment‐rich diets, such as carotenoid sources from alfalfa (*Medicago sativa*) [66], red yeast (*Xanthophyllomyces dendrorhous*) [67], China rose (*Hibiscus rosasinensis*) petals [68], and sweet potato (*Ipomoea batatas* L.) [69]. The study on pigment accumulation in fancy carp demonstrated a proportional increase in carotenoid and anthocyanin levels with higher MRE supplementation. Among the tissues analyzed, the fins showed the highest levels of carotenoid and anthocyanin deposition, highlighting enhanced pigmentation. This tissue‐specific distribution suggests that MRE supplementation effectively enhances carotenoid deposition in regions essential for color expression, thereby improving the visual appeal of ornamental fish. These findings are consistent with the observed increase in chromatophore distribution in goldfish fed diets enriched with microencapsulated anthocyanins [20]. Similarly, Paripatananont et al. [70] documented a 100% increase in chromatophore cells in goldfish provided with a diet containing 100 mg kg^−1^ astaxanthin compared to a control diet without supplementation. Additionally, Xu et al. [67] reported enhanced carotenoid deposition in fantail goldfish following dietary supplementation with astaxanthin.

Histological cross‐sections of the intestine revealed notable increases in villus height in fancy carp fed diets supplemented with MRE‐3. This structural enhancement was directly associated with the improved growth performance observed in fish. The increase in villus height reflects an increased intestinal surface area, which facilitates more efficient nutrient absorption, thereby contributing to better growth rates [71]. Phenolic compounds are known to possess antimicrobial and anti‐inflammatory properties that promote gut health by modulating the gut microbiota and mitigating inflammatory responses in the intestinal lining [72]. These effects create a favorable environment for digestion and nutrient assimilation. Furthermore, phenolic compounds have been shown to stimulate mucin production, reinforcing the intestinal barrier’s integrity and providing protection against pathogens [73]. Comparable benefits of dietary bioactive compounds have been observed in other aquaculture species. For instance, supplementation with mango seed (*Mangifera indica*) powder, rich in phenolic compounds, has been shown to enhance intestinal morphology, including increased villus height and crypt depth, in Nile tilapia, which in turn improves feed utilization and growth performance [74]. Histopathological alterations in the liver were observed across all treatment groups, including pyknotic nuclei, vacuolar degeneration, hydropic swelling, and hyaline degeneration. Notably, fish receiving the highest level of MRE supplementation (4%) exhibited an increased incidence of hydropic swelling, which may represent a hepatic response to elevated concentrations of bioactive compounds. Although these alterations were not overtly pathological, the findings highlight the importance of carefully determining the optimal supplementation level. Previous studies have demonstrated that phenolic compounds, particularly anthocyanins, can exhibit pro‐oxidant properties under certain conditions when consumed in excessive amounts, leading to oxidative stress, mitochondrial dysfunction, and disruption of detoxification pathways [57, 75]. Nevertheless, the absence of severe liver damage in the present study is consistent with prior research, which has reporting hepatoprotective effects of dietary phenolics at appropriate dosages, primarily attributed to their antioxidant activity [56]. The observed improvements in growth, immunity, and pigmentation appear to be mechanistically linked. Enhanced intestinal morphology likely facilitates more efficient nutrient assimilation, while elevated antioxidant enzyme activities protect cells and pigments from oxidative damage. Together, these effects may contribute to improved growth performance and increased pigment deposition, providing a biologically integrated explanation for the benefits of dietary microencapsulated roselle extract (MRE) in fancy carp. This study is among the first to evaluate microencapsulated roselle extract in an ornamental species, addressing the lack of information on optimal inclusion levels in fancy carp. Microencapsulation protects bioactive compounds during feed processing, overcoming limitations of nonencapsulated extracts. An inclusion level of 3% MRE was identified as the optimal dietary level, providing the greatest overall benefits while minimizing undesirable histological changes, thereby highlighting its efficacy and suitability for ornamental aquaculture.

## 5. Conclusion

Dietary supplementation with MRE, especially at the 3% inclusion level, effectively enhanced growth, hematological parameters, immune function, and physiological responses in fancy carp. These results highlight the potential of MRE as a natural and sustainable feed additive to promote health and productivity in aquaculture. For broader applications, future studies should examine the stability of key bioactive compounds during aquafeed processing and elucidate their molecular mechanisms of action in aquatic species. Additionally, further research in other fish species and assessments of fish meat quality are necessary to fully establish the commercial viability of roselle extract in the aquaculture industry.

## Author Contributions

**Supalug Kattakdad**: conceptualization, data curation, formal analysis, funding acquisition, investigation, methodology, project administration, resources, supervision, validation, visualization, writing – original draft, writing – review and editing. **Nittaya Phungam**: investigation. **Suriya Udduang**: visualization. **Sophasith Thammaboud**: writing – review and editing.

## Funding

This work was supported by the Thailand Science Research and Innovation (Grant FF67/P1‐087).

## Conflicts of Interest

The authors declare no conflicts of interest.

## Data Availability

The data that support the findings of this study are available from the corresponding author upon reasonable request.
